# Porosity Engineering of Eggshell-Derived Nanostructured Hydroxyapatite for Ethanol Upgrading: Structural, Textural and Surface Characterization

**DOI:** 10.1021/acsomega.6c08852

**Published:** 2026-10-27

**Authors:** Adilson Claudio Quizunda, Camila Alves Pelicarto Da Silva, Priscila Carvalho Borba Cardoso, Francisco José Moura, Clarissa Perdomo Rodrigues, Marilza Sampaio Aguilar, José Brant De Campos

**Affiliations:** † Graduate Program in Mechanical Engineering Ppg-em, UERJ, São Francisco Xavier Street, 524, Rio de Janeiro, RJ 20550-900, Brazil; ‡ Pontifical Catholic University of Rio De Janeiro (PUC-Rio), Marques De São Vicente Street, 225, São Vicente, 225, Gávea, Rio De Janeiro, RJ 22451-900, Brazil; § Foundation for the Support of the Technical School of Rio De Janeiro (FAETEC), Clarimundo De Melo Street, 847, Quintino Bocaiuva, Rio De Janeiro, RJ 21311-280, Brazil; ∥ Instituto Nacional De Tecnologia (INT), Avenida Venezuela, 82, Room 514, Rio De Janeiro, RJ 20081-312, Brazil

## Abstract

Hydroxyapatite (HAp) has attracted considerable attention as a catalyst owing to its tunable acid–base properties; however, the influence of porogen-assisted synthesis on its textural characteristics and catalytic behavior remains insufficiently understood. In this work, nanostructured HAp was synthesized via a sol–gel route using calcium precursors derived from chicken eggshell waste, combining waste valorization with sustainable catalyst design. The results demonstrate a relationship between porogen-assisted modification of eggshell-derived hydroxyapatite, the resulting textural and acid–base properties, and the relative *n*-butanol yield obtained during relative *n*-butanol yield under the investigated conditions. The incorporation of pore-forming agents modified the textural properties of HAp, increasing the specific surface area from approximately 28 to 155 m^2^ g^–1^ and enhancing the pore volume. Nitrogen adsorption–desorption analyses revealed mesoporous structures with pore sizes ranging from 5 to 35 nm, while the glucose-templated sample exhibited a bimodal pore-size distribution indicative of porosity. The increase in surface area and pore accessibility improved the availability of acid–base active sites, as confirmed by NH_3_-TPD and CO_2_-TPD measurements. A structure–activity relationship was observed, with the most porous catalysts achieving relative *n*-butanol yields above 87%, based on normalized GC peak areas at 180 °C. Product distribution analysis based on gas chromatographic peak areas revealed enhanced relative *n*-butanol yields and improved selectivity toward *n*-butanol formation for catalysts with higher surface areas and mesoporosity. These findings demonstrate that waste-derived eggshells can be effectively converted into porous hydroxyapatite catalysts with tunable textural and acid–base properties, offering a sustainable approach for ethanol upgrading toward *n*-butanol.

## Graphical abstract

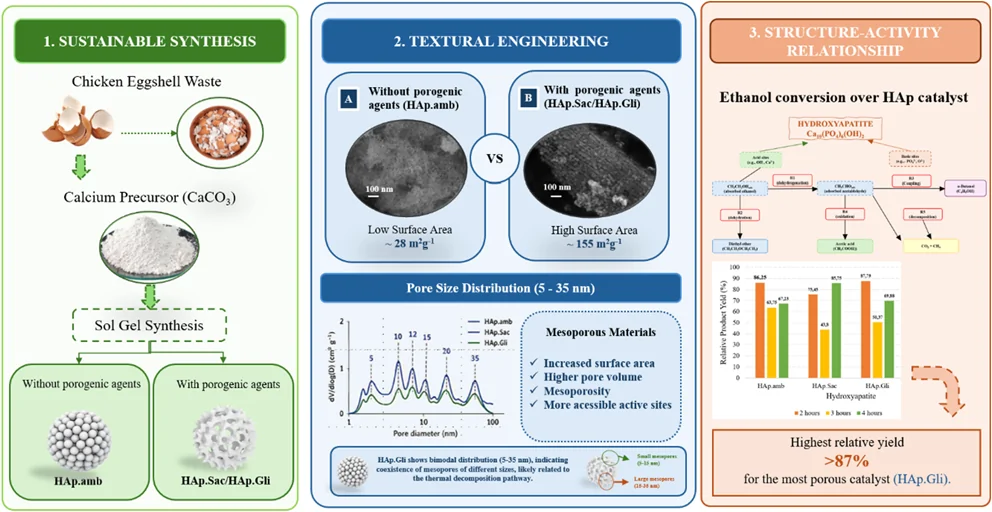

## 1 Introduction

Hydroxyapatite (HAp) is one of the most extensively investigated bioceramic materials owing to its excellent biocompatibility, bioactivity, and close structural resemblance to the mineral phase of human bones and teeth. These characteristics have enabled its widespread use in orthopedic and dental implants, coatings for metallic prostheses, and tissue-engineering applications.^1,2^ Beyond the biomedical field, HAp has attracted considerable attention as a catalyst support, adsorbent, and functional material for controlled-release fertilizers, water treatment, and environmental remediation processes due to its ability to interact with metal ions and organic molecules through surface adsorption mechanisms.^3,4^

The physicochemical properties of HAp are strongly dependent on the synthesis route employed. Methods such as chemical precipitation, hydrothermal synthesis, electrocrystallization, pyrolysis, freeze-drying, microwave-assisted synthesis, and sol–gel processing can significantly influence crystallinity, morphology, particle size, surface area, and pore structure, ultimately affecting the material performance in biomedical and catalytic applications.^5–7^ Among these approaches, the sol–gel method offers remarkable versatility by enabling precise control over composition and microstructure while allowing the use of a broad range of calcium precursors, including waste-derived biological resources such as eggshells and animal bones.^8^ Furthermore, sol–gel processing generally produces materials with high homogeneity, tunable porosity, and enhanced surface area, all of which are desirable characteristics for catalytic applications.^9,10^

The use of waste-derived calcium sources has emerged as an attractive strategy to reduce production costs and improve the sustainability of HAp synthesis.^11,12^ In this context, chicken eggshells represent a particularly promising raw material because they consist predominantly of calcium carbonate (CaCO_3_) and are generated in large quantities worldwide by the food industry. As one of the world’s leading poultry producers, Brazil generates substantial amounts of eggshell waste, which may cause environmental and sanitation issues when inadequately disposed of. Consequently, eggshells have been increasingly investigated as precursors for functional materials and heterogeneous catalysts, contributing to waste valorization and circular-economy strategies.^4^ The conversion of this abundant agro-industrial residue into value-added materials not only mitigates waste-disposal problems but also enhances the environmental sustainability of advanced material production.

Although HAp synthesized from natural resources has been extensively studied for biomedical applications,^3,7^ increasing attention has been devoted to its use in heterogeneous catalysis. The unique acid–base properties of HAp enable its application in reactions such as dehydration and dehydrogenation of alcohols, where catalytic activity is strongly influenced by surface composition and structure.^5^ The growing interest in these applications is closely associated with the ongoing energy transition and the search for sustainable catalytic processes capable of transforming renewable feedstocks derived from biomass.^8,11^

Among biomass-derived molecules, ethanol occupies a strategic position owing to its large-scale production, particularly in Brazil, where sugar cane-based ethanol constitutes a major renewable fuel. In addition to reducing greenhouse-gas emissions relative to fossil fuels, ethanol can serve as a versatile platform molecule for the synthesis of value-added chemicals.^13,14^ One notable example is its conversion into *n*-butanol through the Guerbet reaction, which has attracted significant interest due to the superior fuel properties and broader industrial applicability of higher alcohols.^15,16^ Consequently, the development of efficient and sustainable catalysts for ethanol upgrading remains an important challenge within the framework of green chemistry and the circular bioeconomy.^17,18^

From a structural perspective, hydroxyapatite crystallizes predominantly in a hexagonal lattice, although a monoclinic phase can also be observed under specific conditions. Its composition can be represented by the generalized formula Ca_10–_ *_x_* Na*_x_* M*_x_* (PO_4_)_6–_ *_x_* (CO_3_)*_x_* (OH)_2–_ *_x_*, where M denotes divalent (Mg^2+^, Zn^2+^, Mn^2+^) or trivalent (Al^3+^, Fe^3+^) cations.^19^ Such compositional flexibility allows the modulation of surface acidity, basicity, and catalytic behavior, making HAp an attractive material for heterogeneous catalysis. In addition to chemical composition, textural properties such as specific surface area and pore structure play crucial roles in determining catalyst performance because they control active-site accessibility, reactant adsorption, and mass-transfer phenomena.^20^ Consistent with this relationship, Shen et al. demonstrated that HAp materials possessing higher surface area and well-developed porosity exhibit superior adsorption capacity and enhanced performance in heterogeneous processes.^21^ Therefore, engineering the porous architecture of HAp represents a key strategy for improving catalytic efficiency.^22^

Despite these advantages, the catalytic performance of hydroxyapatite is frequently limited by its inherently low surface area and restricted porosity. Although several synthesis approaches have been explored to overcome these limitations, the development of sustainable and effective strategies for tailoring the pore structure of waste-derived HAp remains insufficiently investigated. In this work, porosity engineering was achieved through the incorporation of glucose and sucrose as sacrificial porogens during the sol–gel synthesis of hydroxyapatite derived from chicken eggshell waste. The controlled decomposition of these organic templates generated mesoporous structures with enhanced surface area and pore connectivity. The relationship between pore architecture and catalytic behavior was systematically evaluated, demonstrating how mesoporosity influences active-site accessibility and mass transport. This study provides a sustainable route for the production of high-performance hydroxyapatite catalysts from agro-industrial waste while advancing the understanding of structure–activity relationships in porous HAp materials.

## 2 Methodology

### 2.1 Catalyst Preparation

White chicken eggshells were employed as the calcium source for hydroxyapatite synthesis. Only white eggshells were selected to obtain stoichiometric HAp, as previously reported.^23,24^ Initially, 3.089g of CaO derived from calcined eggshells were dispersed in 25 mL of distilled water and stirred at 60 °C for 30 min to produce a Ca­(OH)_2_ suspension.^22,25^

Subsequently, 25 mL of a 3.37 mol L^–1^ H_3_PO_4_ solution were added dropwise at a flow rate of 3.33 mL min^–1^. The reactants were calculated to provide a nominal Ca/P molar ratio corresponding to stoichiometric hydroxyapatite. During the reaction, the temperature was maintained at 80 °C and the pH was controlled between 9 and 11 by the addition of NH_4_OH. To modify the textural properties of the material, 3g of solid sucrose or glucose were added during synthesis as sacrificial porogenic agents.^26^ These organic compounds were incorporated into the reaction medium and subsequently decomposed during calcination, generating a network of interconnected pores and promoting an increase in the specific surface area of the resulting hydroxyapatite.^27^

After an aging period of 1 h, the precipitate was filtered, thoroughly washed with distilled water, and dried at 100 °C for 2 h. The dried material was then calcined at 600 °C for 2 h to remove residual organic species and stabilize the porous structure.^28^

The synthesized samples were designated according to the additives employed during preparation. The hydroxyapatite produced without the addition of porogenic agents was denoted as HAp.amb and used as the reference material. Samples prepared in the presence of sucrose and glucose were identified as HAp.Sac and HAp.Gli, respectively. This nomenclature enables a direct comparison of the influence of each porogenic agent on the structural, morphological, textural, and catalytic properties of hydroxyapatite.

A schematic representation of the synthesis procedure is presented in Figure 1, highlighting the main steps involved in the preparation of HAp.amb, HAp.Sac, and HAp.Gli, as well as the role of the porogenic agents in the development of the porous structure.

**Figure 1:**
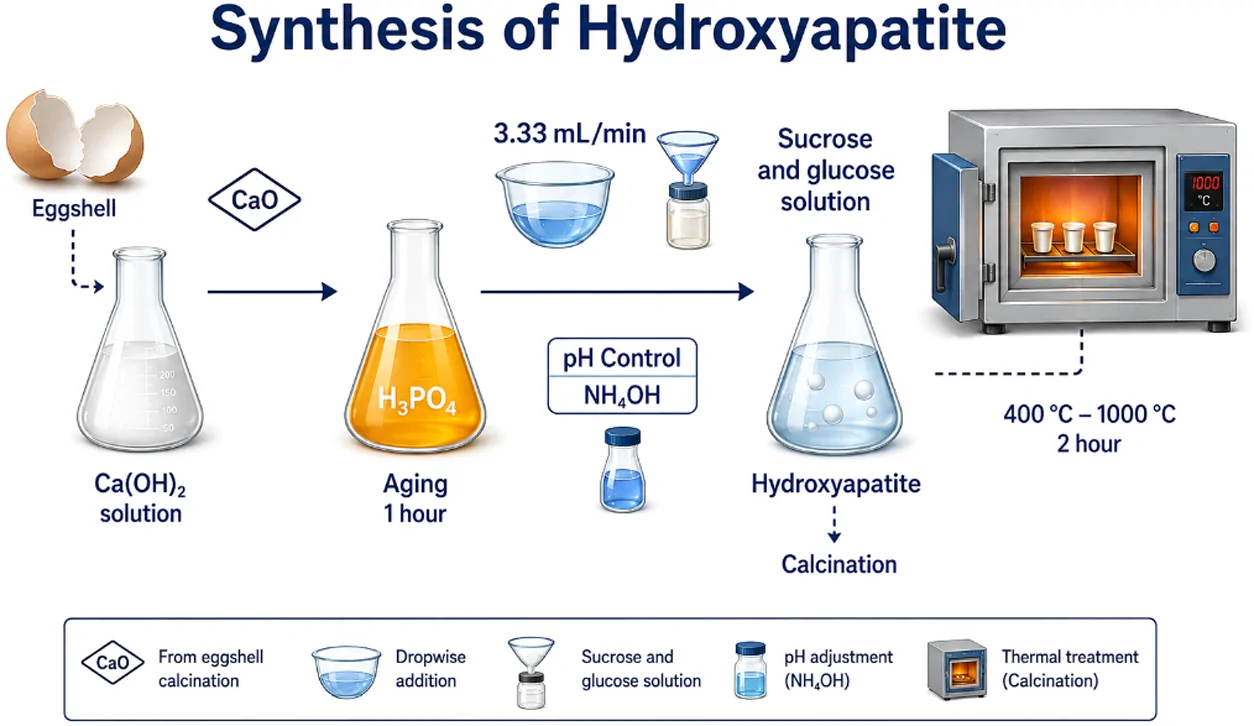
Schematic illustration of the synthesis pathway and catalytic evaluation of eggshell-derived hydroxyapatite materials. The figure was generated with the assistance of artificial intelligence for illustrative purposes only.

## 3 Characterization

### 3.1 Physical Adsorption of N_2_

The nitrogen adsorption–desorption isotherms were taken at −196 °C for the textural characterization of the synthesized materials. The specific surface area was obtained by the Brunauer–Emmett–Teller (BET) method, while pore volume and pore size distribution were taken from Barrett–Joyner–Halenda (BJH) method in a range of 17 to 3000 Å. The average pore diameter was estimated using the 4 V/A ratio, based on BET data.

The analyses were performed at the Department of Chemical and Materials Engineering (DEQM) of PUC-Rio using a Micromeritics ASAP 2010 instrument. The samples were pretreated under vacuum at 300 °C for 12 h to remove mixture and volatile impurities. Isotherms were obtained by the volumetric method, providing information on adsorption capacity, hysteresis, and pore-filling mechanisms associated with the presence of mesoporous and macroporous.

### 3.2 Thermogravimetric Analysis (TGA)

Thermogravimetric analysis was employed to assess the thermal stability and transformations of the synthesized catalysts. Measurements were conducted using a STA 449 F3 Jupiter instrument. Samples were heated in air at a flow rate of 10 mL/min from room temperature (approximately 25 °C) to 1200 °C at a constant heating rate of 15 °C/min, followed by a 4 h isothermal hold at the maximum temperature. This procedure enabled the identification of mass loss events associated with dehydration, decomposition of organic residues, and structural alterations within the hydroxyapatite framework. The analysis was performed at DEQM, PUC-Rio.

### 3.3 X-ray Diffraction (XRD)

The characterization of phase formation was performed using XRD (X-ray diffraction) techniques. Measurements were carried out on these samples using a Panalytical Empyrean diffractometer with Cu Kα radiation, a scan step of 0.05°, and an acquisition rate of 2.5°/min. Quantitative calculations of the Rietveld Fundamental Parameters (FP)^29–31^ were performed to determine the phases. The scale factor (s) in the refinement equation correlates directly with the weight fraction of each phase, allowing the quantification of phases in weight percent (wt %). For these calculations, the lattice parameter, crystallite size, and scale factor were adjusted, with the scale factor indicating the weight percent of each phase.^29^

### 3.4 Fourier Transform Infrared Spectroscopy (FTIR)

The HAp samples were dried in an oven, mixed with spectroscopic-grade KBr, and pressed into thin self-supporting pellets for infrared analysis. Spectra were recorded in the range of 400–4000 cm^–1^ with a PerkinElmer Spectrum Two spectrometer at a resolution of 4 cm^–1^. Measurements were taken at the Department of Chemical and Materials Engineering (DEQM) of the Pontifical Catholic University of Rio de Janeiro (PUC-Rio).

### 3.5 Scanning Electron Microscopy (SEM)

Scanning electron microscopy (SEM) was employed to analyze the morphology and surface of hydroxyapatite catalysts, allowing correlation of structural characteristics with catalytic performance. Images were obtained at the Multi-User Laboratory for Nanofabrication and Characterization of Nanomaterials (NANOFAB) at UERJ, using a JEOL JSM-7100F field emission microscope. For sample preparation, the powder was dispersed on double-sided carbon tape, ensuring good fixation and minimizing loading effects. The micrographs allowed evaluation of particle size distribution, surface texture, and the presence of pores or agglomerates.

### 3.6 Determination of Acidic and Basic Sites by TPD (CO_2_ AND NH_3_)

The density and strength of the acidic and basic surface sites of the oxides were determined by temperature-programmed desorption (TPD), using CO_2_ and NH_3_ as probe molecules. The analyses were performed in a microreactor coupled to the Micromeritics AutoChem II 2920 system, at the Laboratory of Catalysis (LACAT/DICAP/INT).

Approximately 100 mg of sample were pretreated under a helium flow (50 mL·min^–1^) at 180 °C for 60 min. Adsorption was performed separately at room temperature for 60 min (50 mL·min^–1^), followed by purging with helium (30 mL·min^–1^) for 30 min. Desorption was conducted under a helium flow (50 mL·min^–1^), from room temperature to 300 °C, monitored by a thermal conductivity detector (TCD), maintaining the final temperature for 90 min. The profiles obtained allowed quantification of the density of surface sites and evaluation of the distribution of acid and base strengths.

The amounts of acidic and basic sites were quantified from NH_3_-TPD and CO_2_-TPD profiles, respectively. Peak areas were obtained from the TCD signal after baseline correction and converted into the amount of desorbed probe molecules using the instrument calibration factor. The resulting values were expressed as mmol of probe molecule per gram of catalyst (mmol g^–1^).

Peak deconvolution was performed using Gaussian fitting to distinguish adsorption sites of different strengths. Low-temperature peaks were assigned to weak sites, intermediate-temperature peaks to medium-strength sites, and high-temperature peaks to strong sites. The total density of acid and base sites was calculated as the sum of all deconvoluted peaks.

### 3.7 Catalytic Tests

Catalytic experiments were carried out in a stainless-steel tubular reactor with an internal volume of 30 mL. The hydroxyapatite catalyst (HAp) and the reaction mixture were introduced directly into the reactor without prior stirring. The reactor was subsequently placed in a programmable temperature-controlled furnace (Furnace Brand), equipped with a heating ramp of 10 °C min^–1^ until the target temperature was reached, ensuring stable thermal conditions throughout the reaction.

The reactions were conducted under solvothermal conditions using 10 mL of ethanol (97% purity), 10 mL of distilled water, and 0.319g of catalyst. The reaction mixture was transferred to a hermetically sealed stainless-steel autoclave and heated to 180 °C under autogenous pressure for 2, 3, or 4 h, depending on the experimental condition investigated.^32^

At 180 °C, the closed reactor operates under the pressure generated by the vapor–liquid equilibrium of the ethanol–water mixture. The autoclave employed in this study was designed for high-temperature and high-pressure operation, allowing the reaction to be safely conducted under the autogenous pressure developed during heating.

Water was employed as the reaction medium to improve phase homogeneity and facilitate contact between the reactants and the catalyst. Under these conditions, ethanol underwent catalytic transformation, producing a mixture of liquid products in which *n*-butanol was identified among the major reaction products, together with other compounds formed through parallel and consecutive reaction pathways.^33,34^

Reaction products were identified by gas chromatography. The values reported throughout this study represent relative product yields derived from chromatographic area normalization rather than absolute molar yields.

The use of relative product yields allowed a direct comparison of catalyst performance and product distribution under identical reaction conditions. This approach was applied consistently throughout the manuscript for the evaluation of the hydroxyapatite-based catalysts.

All catalytic experiments were under identical reaction conditions. Ethanol conversion, *n*-butanol selectivity, and relative *n*-butanol yield were calculated from gas chromatographic analyses, and the results are reported as mean values. Statistical comparisons among samples were performed using one-way analysis of variance, with differences considered statistically significant at *p* < 0.05.

Figure 2 summarizes the experimental procedure employed for the solvothermal upgrading of ethanol over hydroxyapatite catalysts, highlighting the main steps performed in the stainless-steel reactor at 180 °C under autogenous pressure.

**Figure 2:**
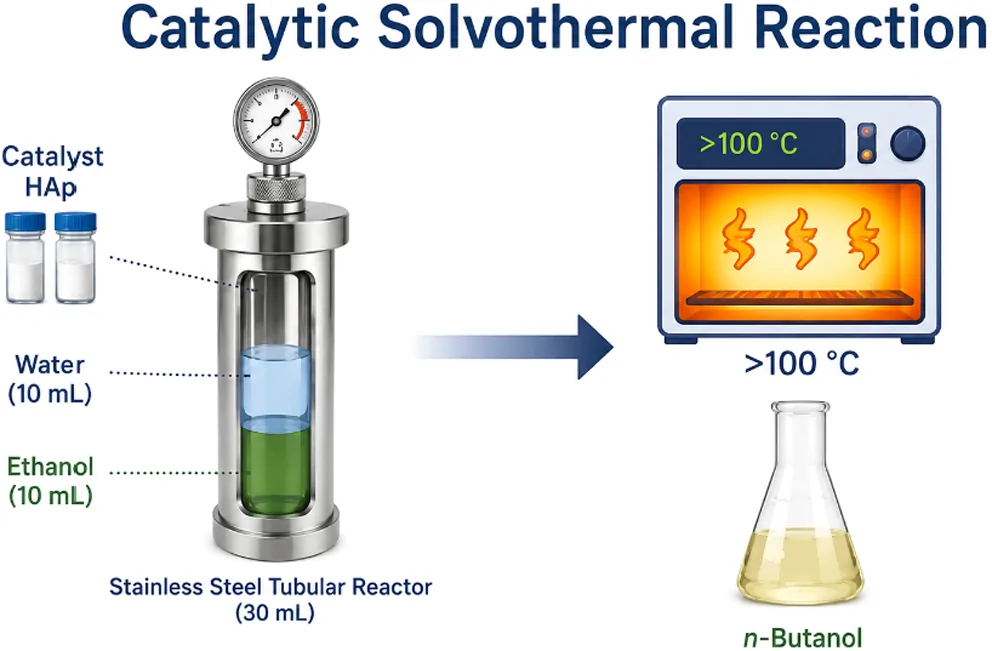
Solvothermal conversion of ethanol to *n*-butanol catalyzed by hydroxyapatite (HAp) in a stainless-steel tubular reactor under autogenous pressure and a temperature above 100 °C. Illustration generated using AI and reviewed by the authors.

## 4 Results and Discussion

### 4.1 Physical Adsorption of N_2_

The textural properties of the hydroxyapatite samples calcined at 600 °C, namely HAp.amb (without porogenic agent), HAp.Sac (sucrose-modified), and HAp.Gli (glucose-modified), were investigated by nitrogen adsorption–desorption measurements at 77 K. Prior to analysis, all samples were degassed under vacuum to remove physically adsorbed species. The specific surface area was determined using the Brunauer–Emmett–Teller (BET) method from adsorption data collected within the appropriate relative pressure range (*P*/*P*_0_), while the total pore volume was estimated from the amount of nitrogen adsorbed near saturation pressure. Pore-size distributions were calculated using the Barrett–Joyner–Halenda (BJH) method applied to the desorption branch of the isotherms.

The adsorption–desorption isotherms revealed significant differences in the textural properties of the synthesized materials, indicating that the incorporation of porogenic agents markedly influenced the porous structure of hydroxyapatite. Both sucrose and glucose promoted the formation of mesoporous networks, resulting in substantial increases in specific surface area and pore volume compared with the pristine material (HAp.amb).

The textural parameters summarized in Table 1 confirm the effectiveness of these additives in tailoring the porous architecture of hydroxyapatite. Relative to HAp.amb, the specific surface area increased by approximately 5.5-fold for HAp.Sac and 4.5-fold for HAp.Gli. These results demonstrate that the use of porogenic agents during synthesis significantly modifies the surface and pore characteristics of the material, generating structures that may favor reactant accessibility and diffusion during catalytic reactions.

**Table 1:** Textural Properties of Synthesized Samples

Samples	BET Specific Surface (m^2^/g)	Average pore size (Å)	Pore volume (cm^3^/g)
HAp.amb	28	16.2	0.147
HAp.Sac	155	154.6	0.598
HAp.Gli	125	74.2	0.594

Figure 3 presents the N_2_ adsorption–desorption isotherms of the HAp.amb, HAp.Sac, and HAp.Gli samples. The HAp.amb sample exhibited a Type II isotherm, characterized by relatively low N_2_ uptake and the absence of a significant hysteresis loop, suggesting limited pore development and reduced accessibility to the internal surface. In contrast, the HAp.Sac and HAp.Gli samples displayed Type IV isotherms with pronounced hysteresis loops, indicating the presence of mesoporous structures.^35–37^ The change in isotherm profile from Type II to Type IV highlights the effect of the synthesis additives on pore formation and textural evolution. In particular, HAp.Sac exhibited the highest adsorbed volume at elevated relative pressures, indicating a more extensive and interconnected mesoporous network. Although HAp.Gli also displayed characteristics typical of mesoporous materials, its lower adsorbed volume relative to HAp.Sac suggests a lower degree of pore development and intermediate textural properties.^36^

**Figure 3:**
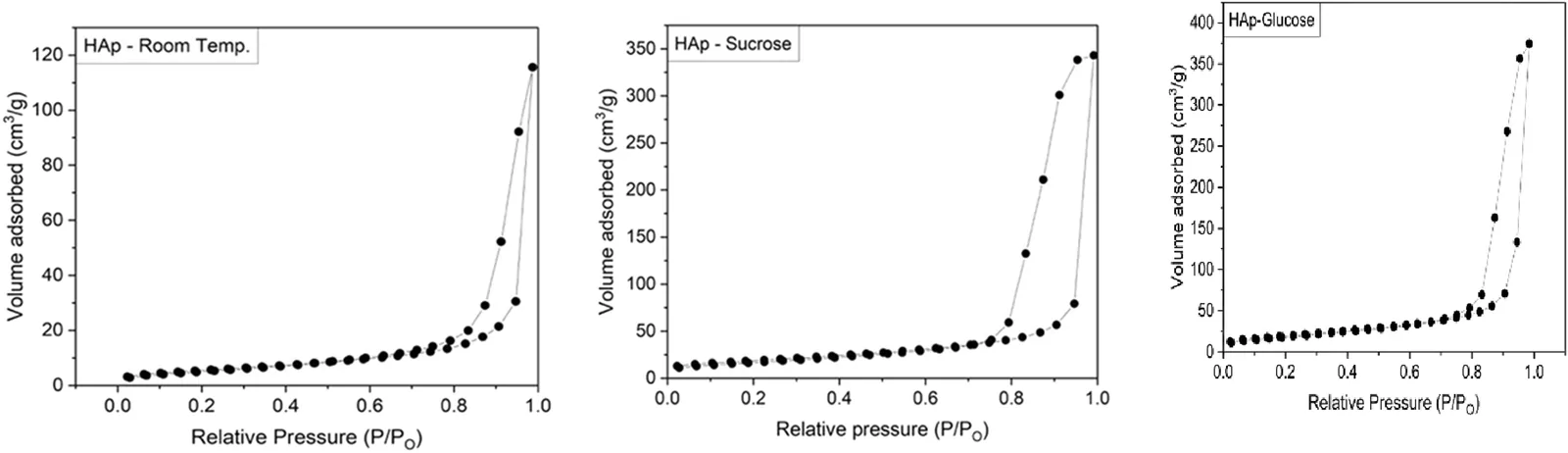
Nitrogen adsorption/desorption isotherms of samples HAp.amb, HAp.Sac, and HAp.Gli.

In contrast, HAp.Sac and HAp.Gli displayed substantially higher adsorption capacities and more evident hysteresis loops, characteristic of well-developed mesoporous structures. Among the modified materials, HAp.Sac exhibited the highest adsorption volume throughout the relative pressure range, suggesting the formation of a more extensive and interconnected mesopore network. HAp.Gli also showed mesoporous behavior, although with lower adsorption capacity than HAp.Sac, indicating an intermediate degree of pore development.^38^

The textural parameters further corroborate these observations, revealing higher specific surface area and pore volume values for the sugar-modified samples, particularly HAp.Sac, as shown in Figure 4. These findings demonstrate that the incorporation of pore-forming agents effectively enhances the porosity of hydroxyapatite, which may improve its performance in catalytic applications.

**Figure 4:**
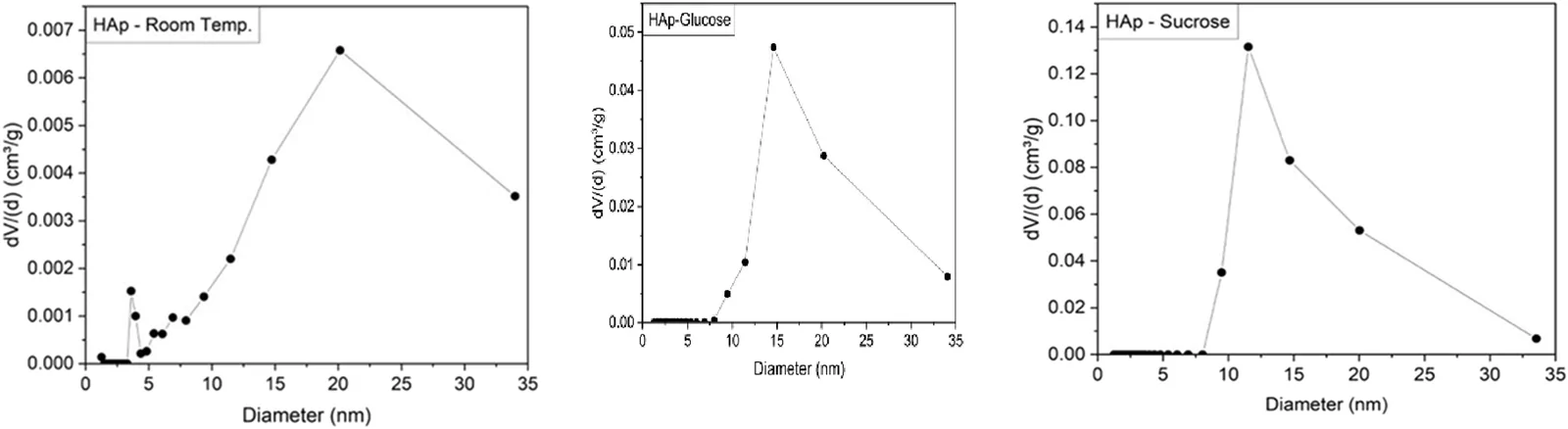
Distribution of mesopore size (5–35 nm) determined by the Barrett, Joyner, and Halenda (BJH) method.

All materials exhibited pore size distributions within the mesoporous range (5–35 nm). Characteristic maxima were observed at approximately 5, 10, 12, 15, 20, and 35 nm for the HAp.amb, HAp.Sac, and HAp.Gli samples. Notably, HAp.Gli presented a bimodal pore size distribution, indicating the coexistence of mesopores with distinct dimensions. This behavior may be associated with the decomposition pathway of glucose during heat treatment, which can promote the formation of pore populations with different sizes.^39^

The presence of well-defined mesoporosity is advantageous for both biomedical and catalytic applications because it facilitates mass transport and increases the available surface for interaction with chemical species. Accordingly, HAp.Sac and HAp.Gli, which exhibited higher surface areas and pore volumes, are promising candidates for applications such as drug delivery systems and heterogeneous catalysis.^40^

Furthermore, all samples exhibited relatively high specific surface area values (28–155 m^2^ g^–1^), exceeding those commonly reported for mesoporous calcium phosphate materials. For instance, sucrose-assisted systems generally achieve surface areas of approximately 90 m^2^ g^–1^.^41^ These results highlight the effectiveness of sucrose and glucose as porogenic agents for generating highly porous hydroxyapatite structures through a simple, low-cost, and environmentally friendly synthesis strategy. Overall, the use of sugars as pore-forming agents significantly improved the textural properties of hydroxyapatite, broadening its potential for advanced technological applications.^42^

### 4.2 Thermogravimetric Analysis (TGA)

Thermogravimetric analysis (TGA) was used to monitor the thermal decomposition of the porogenic agents and to evaluate the thermal stability of the hydroxyapatite samples (HAp.Amb, HAp.Sac, and HAp.Gli) during calcination at 600 °C. This temperature is sufficient to ensure the complete removal of carbonaceous residues derived from sucrose and glucose, as previously reported in the literature. Figures 5, 6, and 7 present the TGA curves for the samples synthesized with the addition of 3g of sucrose or glucose and subsequently heated in synthetic air for 4 h.

**Figure 5:**
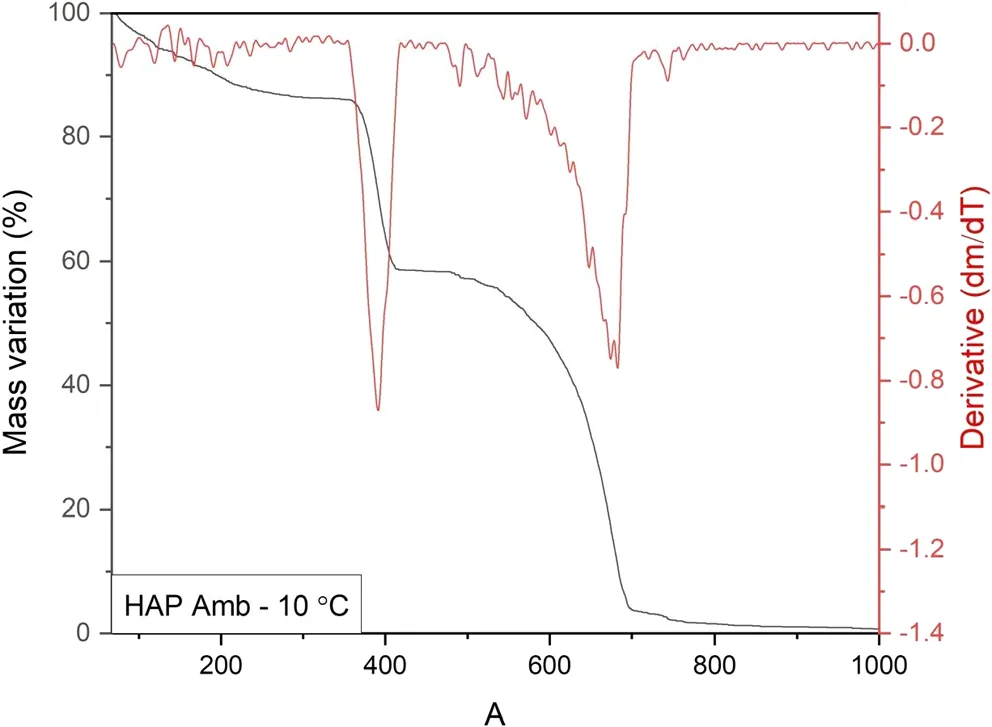
Thermogravimetric analysis of HAp.Amb.

**Figure 6:**
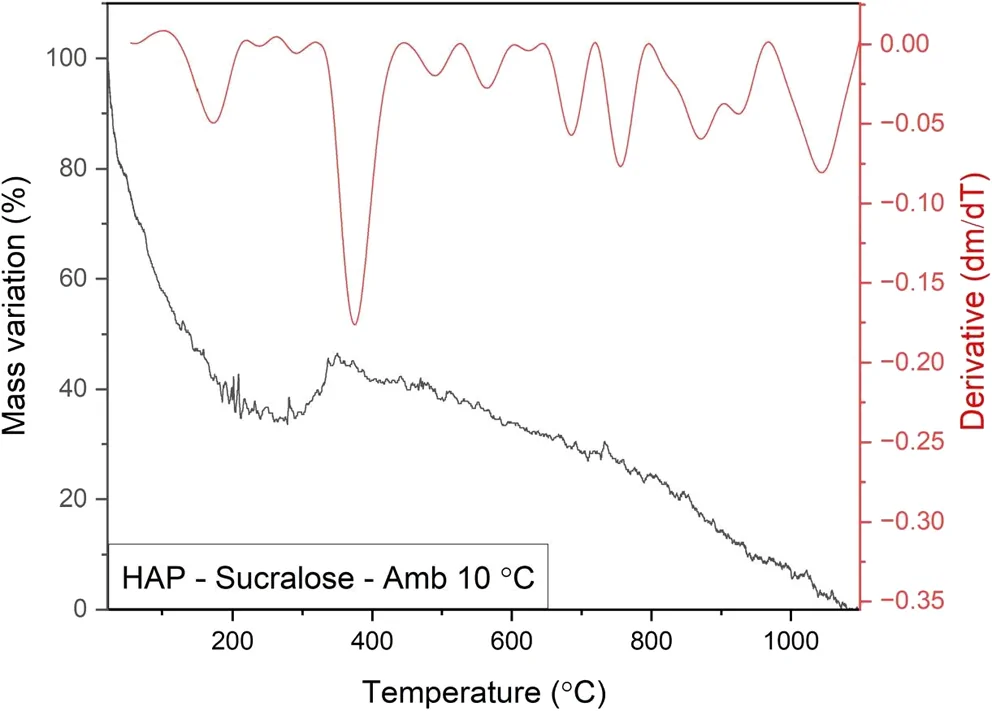
Thermogravimetric analysis of the HAp.Sac.

**Figure 7:**
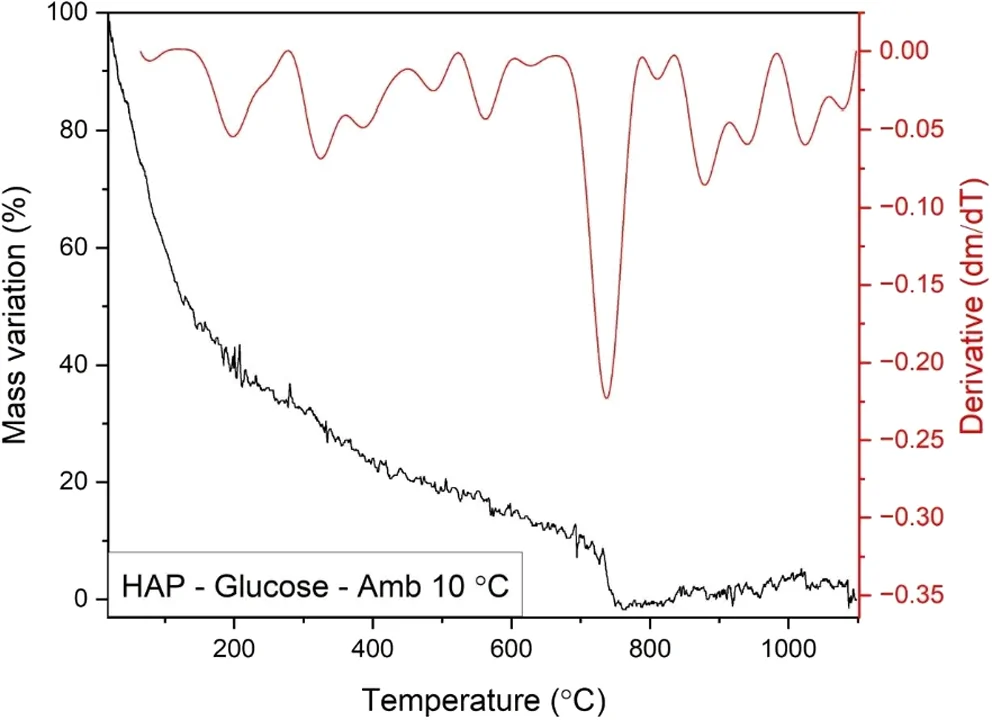
Thermogravimetric analysis of the HAp.Gli.

The TGA profiles reveal multiple mass loss events. A minor initial loss below 200 °C corresponds to the desorption of physically adsorbed water. The major mass loss region, occurring between 200 and 600 °C, is associated with the degradation and oxidation of the organic porogenic agents. Above 600 °C, the curves reach a plateau, indicating that carbonaceous species have been fully removed and that the remaining material consists predominantly of inorganic hydroxyapatite. All samples whether containing porogenic additives or not exhibited negligible mass variation up to 1000 °C, confirming the high thermal stability and structural robustness of the hydroxyapatite framework.

For the sucrose and glucose containing samples, the more pronounced mass loss steps observed in the TGA curves confirm the effective incorporation and subsequent thermal decomposition of the porogenic agents. Complete removal of these organics is essential to ensure that the increases in surface area and porosity are not accompanied by residual carbon, which could compromise catalytic performance or biocompatibility.^51^ Overall, the TGA results demonstrate that calcination at 600 °C is adequate not only for eliminating organic porogens but also for producing thermally stable mesoporous hydroxyapatite suitable for catalytic applications.

### 4.3 X-ray Diffraction (XRD)

X-ray diffraction (XRD) was employed to identify the crystalline phases and estimate the average crystallite size of the samples calcined at 600 °C.^43^ The crystallite sizes obtained from the Rietveld refinement, together with the corresponding goodness-of-fit (GOF) values, are presented in Table 2. The GOF values, ranging from 1.39 to 1.61, indicate satisfactory agreement between the experimental and calculated diffraction patterns, confirming the reliability of the refined structural parameters.

**Table 2:** Crystallite Size and GOF Values of HAp Samples (Rietveld Refinement)

	Average crystallite size(nm)	
Sample	HAp/ β-TCP	GOF
HAp.amb	13.82	1.61
HAp.Sac	11.50	1.39
HAp.Gli	12.23	1.49

The HAp.amb, HAp.Sac, and HAp.Gli samples exhibited average crystallite sizes within the range of 11–14 nm, indicating that the incorporation of sucrose and glucose did not significantly affect crystal growth during calcination.^44^ These results suggest that the porogenic agents primarily influenced the textural properties of the materials while preserving the crystalline characteristics of hydroxyapatite. The corresponding XRD patterns and Rietveld refinements are shown in Figure 8.

**Figure 8:**
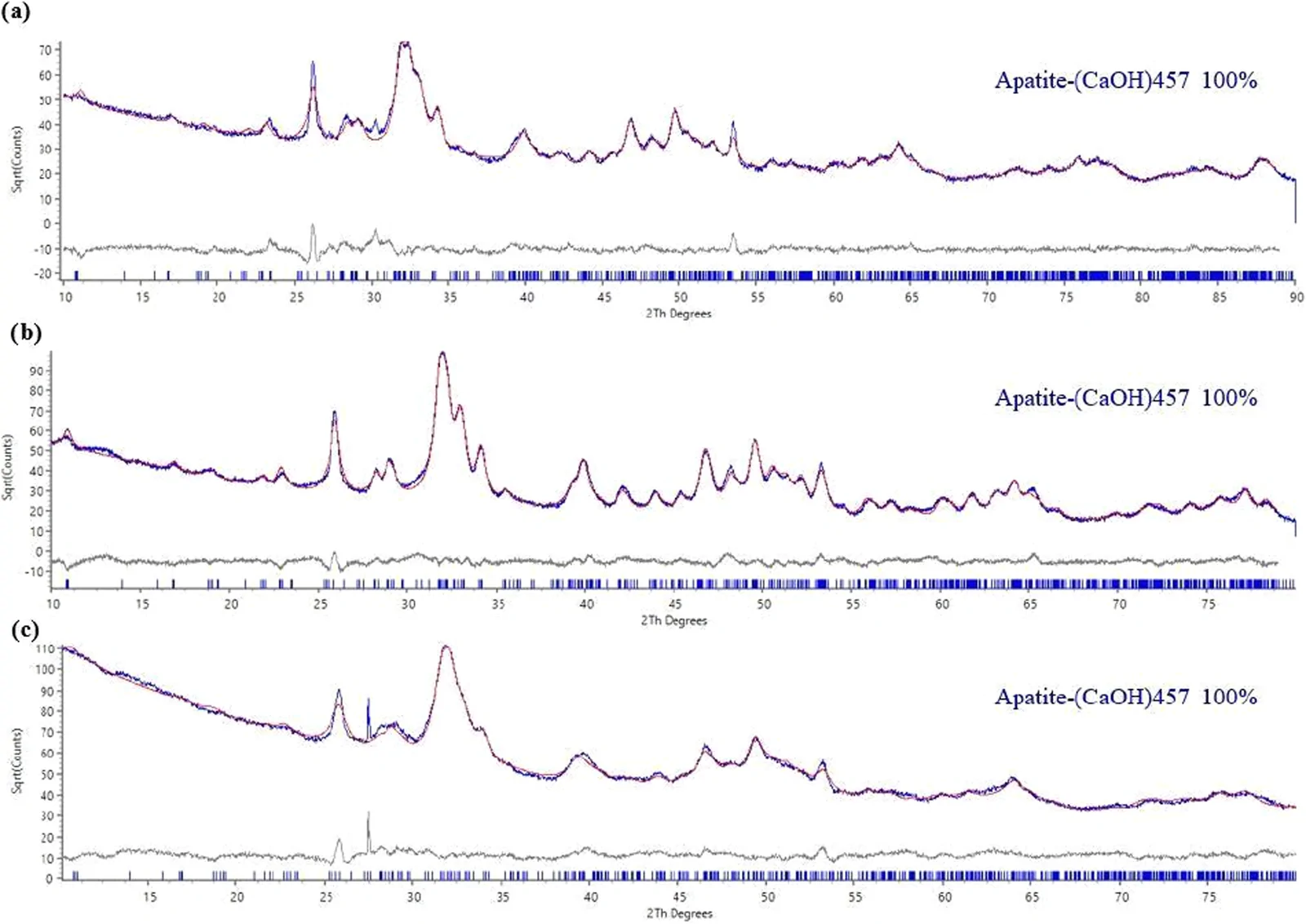
Diffractogram obtained from the Rietveld method refinement of the (a) HAp.amb sample (b) HAp.Sac sample and (c) HAp.Gli sample.

XRD analyses were performed to evaluate the phase composition of all samples, and quantitative phase analysis was conducted using the Rietveld refinement method. In Figure 5, the blue curves correspond to the experimental diffraction data, whereas the red curves represent the calculated patterns obtained from the refinement. The experimental diffractograms exhibited a favorable signal-to-noise ratio, contributing to the high quality of the refinements and the low GOF values obtained for all samples. The gray curves correspond to the difference between the observed and calculated patterns. The small residual intensity observed throughout the refinement indicates excellent agreement between the experimental and simulated data, supporting the reliability of the phase identification and quantification.

The X-ray diffraction patterns revealed the exclusive presence of the hydroxyapatite phase in all samples, with no detectable secondary crystalline phases. Rietveld refinement confirmed a phase purity of 100% hydroxyapatite for HAp.amb, HAp.Sac, and HAp.Gli. Phase identification and structural refinement were performed using ICSD entry 34457 as the reference phase. The refinement was carried out using a monoclinic hydroxyapatite structural model (space group *P*11_2_1/*b*), yielding lattice parameters of *a* = 9.492 Å, *b* = 18.831 Å, *c* = 6.880 Å, and γ = 120°. These values are consistent with the monoclinic crystallographic form of hydroxyapatite frequently reported for materials derived from natural and biomimetic sources.^29^

The crystallite sizes determined by Rietveld refinement were 13.82, 11.50, and 12.23 nm for HAp.amb, HAp.Sac, and HAp.Gli, respectively. These values confirm the nanocrystalline nature of the synthesized materials and indicate that the use of different porogenic agents did not induce substantial changes in crystalline phase composition. Instead, the primary effect of sucrose and glucose addition was associated with modifications in the porous structure, as demonstrated by the nitrogen adsorption–desorption analyses discussed previously.

### 4.4 Fourier Transform Infrared Spectroscopy (FTIR)

Figure 9 presents the FTIR spectra of the hydroxyapatite samples synthesized in the presence of 3g of sucrose or glucose and subsequently calcined at 600 °C. The spectra display the characteristic vibrational modes of phosphate, hydroxyl, and carbonate groups, confirming the successful formation of the hydroxyapatite phase. In addition, subtle differences among the spectra suggest that the porogenic agents influenced the surface chemical properties of the obtained materials.

**Figure 9:**
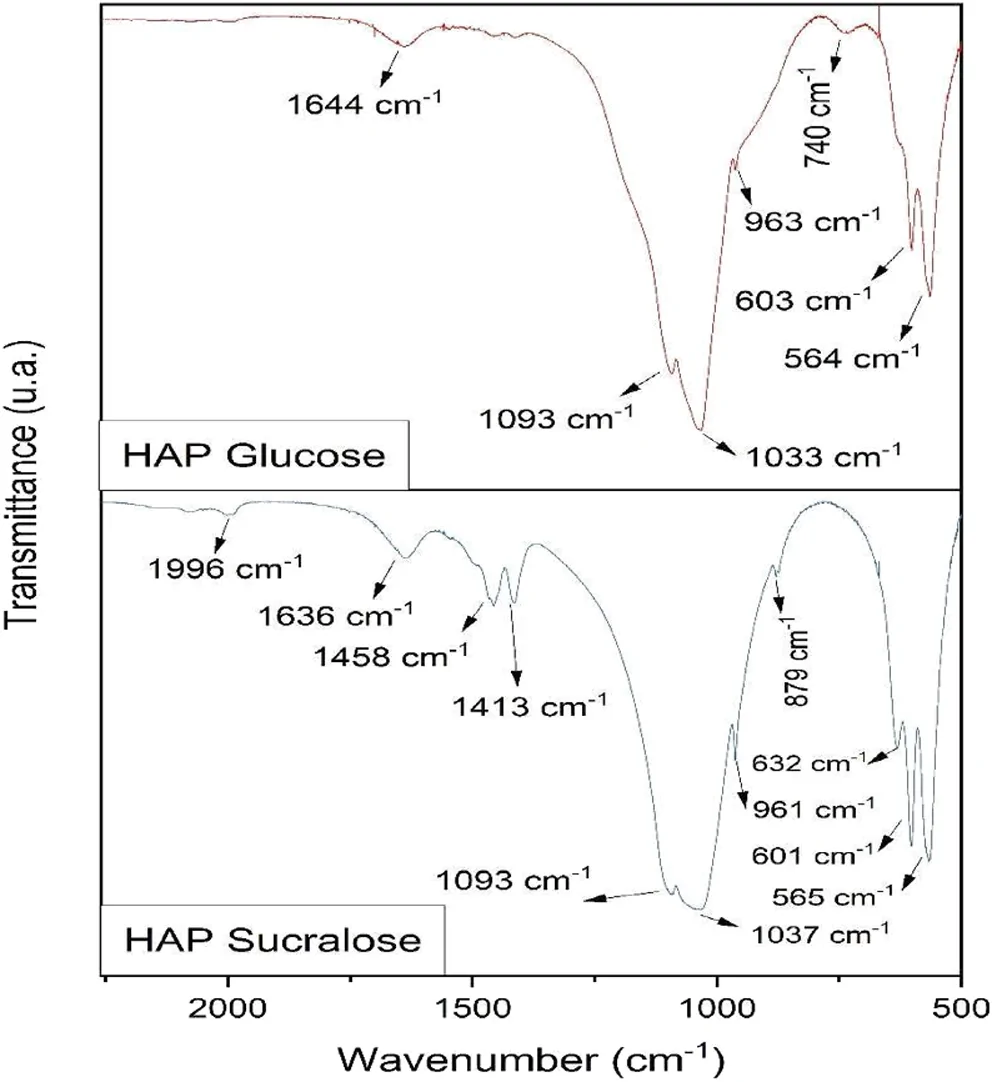
FTIR of calcined samples at 600 °C.

All samples exhibited the characteristic absorption bands of hydroxyapatite. The bands located at approximately 1093, 1033–1037, and 961–963 cm^–1^ are assigned to the ν_3_ and ν_1_ stretching vibrations of the phosphate (PO_4_^3–^) groups, whereas the bands observed at approximately 603–601 and 565–564 cm^–1^ correspond to the ν_4_ bending modes of phosphate, indicating the formation of a well-defined apatite framework.^33,34^ The absorption band centered around 1640 cm^–1^ is attributed to the bending vibration of adsorbed water molecules on the material surface.

For the HAp.Sac sample, additional absorption bands in the regions of 1458–1413 and approximately 879 cm^–1^ were observed, which are characteristic of carbonate species. These bands suggest partial carbonate incorporation into the hydroxyapatite structure, possibly through ionic substitution, which may influence the surface acid–base properties and, consequently, the catalytic behavior of the material.^10,27^

Notably, no absorption bands associated with C–H stretching vibrations (2800–3000 cm^–1^) or carbonyl groups (∼1700 cm^–1^) were detected in any of the calcined samples. This observation confirms the effective removal of the organic templates during calcination and demonstrates that sucrose and glucose acted as sacrificial pore-forming agents. Therefore, the FTIR results indicate that the incorporation of sugars successfully promoted pore generation while preserving the chemical integrity of the hydroxyapatite structure and maintaining the surface functional groups relevant for catalytic applications.

Combined with the N_2_ adsorption–desorption results, the FTIR analysis demonstrates that the use of sucrose and glucose enables the development of highly porous hydroxyapatite materials without significant alterations to their crystalline framework or surface functional groups, which is advantageous for catalytic processes requiring high surface accessibility and structural stability.

### 4.5 Scanning Electron Microscopy (SEM)

Figure 10 presents the morphological characteristics of the HAp.amb, HAp.Sac, and HAp.Gli samples. The HAp.amb sample exhibits a dense and highly agglomerated morphology, with limited distinction between individual particles, which is typical of hydroxyapatite synthesized without the use of pore-forming agents. In contrast, the HAp.Sac and HAp.Gli samples display a less compact arrangement, consisting of more dispersed and clearly defined particles with rod-like or needle-like morphologies, commonly reported for nanostructured hydroxyapatite materials.^45^

**Figure 10:**
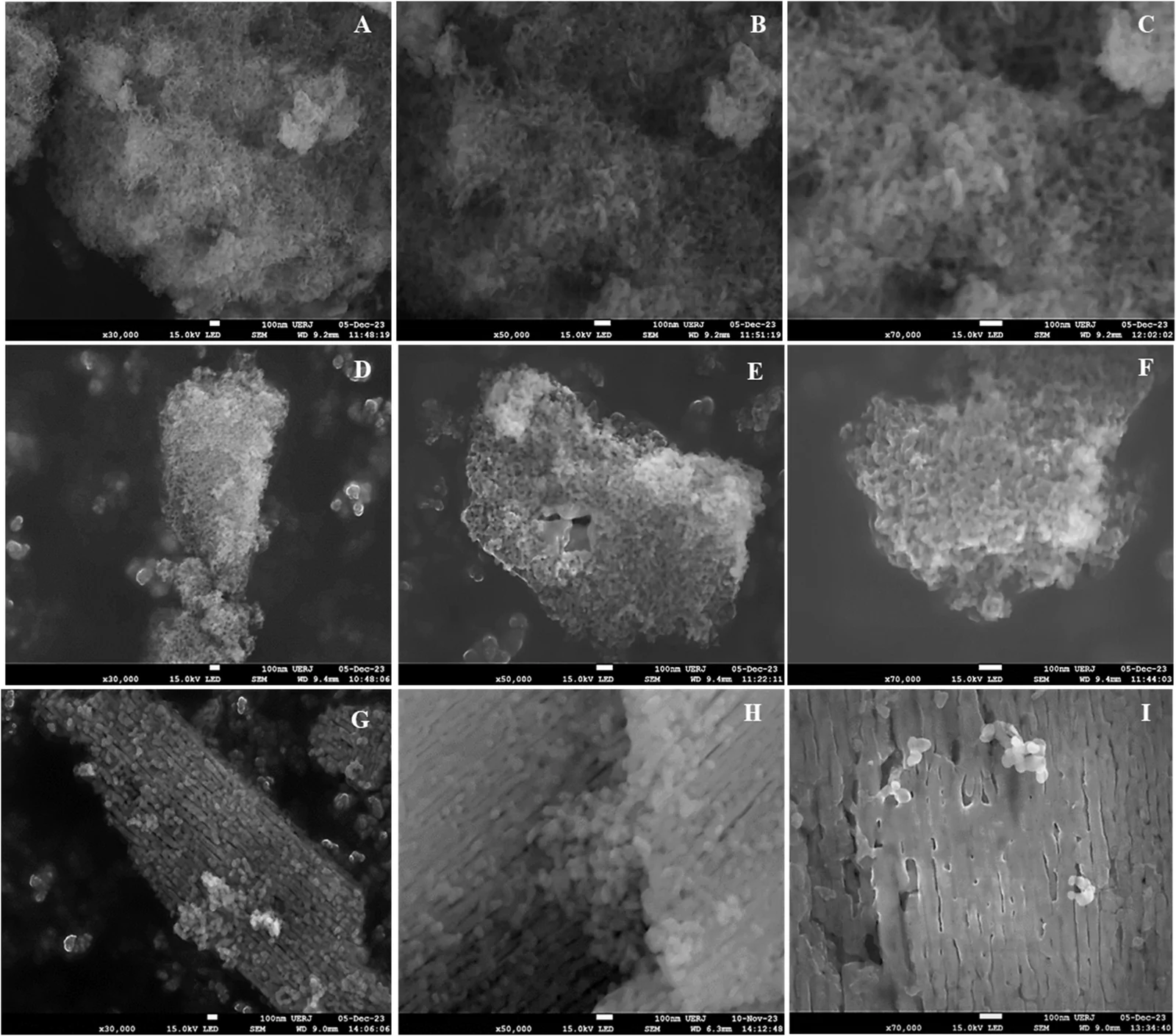
Micrographs of samples HAp.amb (A–C), HAp.Sac (D–F), and HAp.Gli (G–I) at magnifications of 30,000× (A, D, G), 50,000× (B, E, H), and 70,000× (C, F, I).

The modified samples exhibited similar particle dimensions and morphological features, as evidenced by the identical scale bars used in the micrographs. The observed elongated morphology is comparable to that reported for biologically derived apatite structures and indicates that the incorporation of sucrose and glucose during synthesis did not adversely affect the formation of the hydroxyapatite crystalline framework. Instead, these additives promoted structural organization while contributing to pore generation.

A comparison of the micrographs reveals that the addition of porogenic agents reduced particle agglomeration and promoted the formation of more accessible interparticle voids. This observation is consistent with the textural results obtained from N_2_ adsorption–desorption analyses, which demonstrated increased surface area and pore volume for HAp.Sac and HAp.Gli. Although the overall particle morphology remained largely unchanged, the development of a more open and porous nanostructure is evident in the sugar-modified samples.

The combination of nanometric particle morphology, enhanced porosity, and higher surface area is expected to improve mass transport and facilitate access to active surface sites. Consequently, the HAp.Sac and HAp.Gli materials are particularly promising for applications that require efficient surface interactions, including heterogeneous catalysis, adsorption processes, and biomedical systems. Overall, the SEM observations corroborate the textural characterization results, demonstrating that sucrose and glucose act as effective porogenic agents for tailoring the microstructure of hydroxyapatite without compromising its characteristic morphology.

### 4.6 Determination of Acidic and Basic Sites by TPD (CO_2_ AND NH_3_)

Temperature-programmed desorption (TPD) analyses using CO_2_ and NH_3_ were performed to evaluate the distribution and strength of the basic and acidic surface sites of the hydroxyapatite samples. The results demonstrate that the incorporation of sucrose and glucose significantly modified the acid–base properties of the materials, which may directly influence their catalytic behavior.

Quantitative analysis of the NH_3_-TPD and CO_2_-TPD profiles revealed distinct acid–base characteristics among the synthesized hydroxyapatite samples. The calculated densities of acidic and basic sites are summarized in Table 3.

**Table 3:** Acid and Basic Site Densities of the Catalysts Determined by NH_3_-TPD and CO_2_-TPD

Catalyst	Acid Sites (μmol NH_3_g^–1^)	Basic Sites (μmol CO_2_g^–1^)
HAp.amb	101	104
HAp.Sac	100	106
HAp.Gli	100	99

The pristine HAp sample exhibited an acidity of 101 μmol NH_3_ g^–1^ and a basicity of 104 μmol CO_2_ g^–1^. Following modification with sucrose, the resulting HAp.Sac material showed an acidity of 100 μmol NH_3_ g^–1^ and a basicity of 106 μmol CO_2_ g^–1^. In contrast, HAp.Gli presented an acidity of 100 μmol NH_3_ g^–1^ and a basicity of 99 μmol CO_2_ g^–1^. These results indicate that the surface acid–base properties were only slightly affected by the modifications, with HAp.Sac exhibiting the highest basicity and HAp.Gli the lowest

These differences in surface chemistry may explain the catalytic behavior observed during ethanol upgrading. The HAp.Gli catalyst, which exhibited the highest relative *n*-butanol yield, also showed the most favorable balance between acidic and basic surface sites. Such a balance is considered beneficial for the sequential steps involved in the Guerbet reaction, where basic sites promote ethanol dehydrogenation, while cooperative acid–base interactions facilitate aldol condensation and subsequent C–C bond formation. Conversely, the predominance of acidic sites in HAp.Sac may have contributed to its lower relative *n*-butanol yield under the investigated reaction conditions.

Overall, the NH_3_-TPD and CO_2_-TPD results suggest that tailoring the acid–base properties of hydroxyapatite through the use of different porogenic agents significantly influences catalytic performance and product distribution.

The HAp.amb sample exhibited a predominance of medium-strength basic sites, which can be associated with exposed O^2–^ species present in the hydroxyapatite lattice.^42^ The HAp.Gli sample showed a slight increase in the proportion of weak basic sites while maintaining a high percentage of medium-strength sites, suggesting enhanced exposure of surface hydroxyl species. In contrast, HAp.Sac exhibited a markedly different distribution, characterized by a greater proportion of weak basic sites and the appearance of strong basic sites. This behavior may be related to structural modifications induced by the thermal decomposition of sucrose during calcination, which can generate additional surface defects and alter the local chemical environment.^45^

Table 4 summarizes the relative distribution of acidic and basic sites obtained from the deconvolution of the TPD profiles.

**Table 4:** Quantification of Basic and Acidic Sites Identified by TPD, Expressed as Relative Percentage

	BASIC SITES			ACIDIC SITES		
Sample	Weak Sites	Medium Sites	Strong Sites	Weak Sites	Medium Sites	Strong Sites
HAp.amb	30.0%	70.0%	–	10.9%	19.2%	69.9%
HAp.Gli	32.3%	67.7%	–	26.2%	40.5%	33.3%
HAp.Sac	49.6%	22.6%	27.8%	29.1%	70.9%	-

Regarding the acidic properties, HAp.amb exhibited a strong predominance of high-strength acid sites. These sites are commonly associated with phosphate groups (PO_4_^3–^) and Brønsted acidic species related to structural hydroxyl groups of hydroxyapatites.^27^ Compared with the unmodified sample, HAp.Gli displayed a more balanced distribution of acidity, with increased proportions of weak and medium-strength acid sites accompanied by a substantial reduction in strong acid sites. This behavior suggests that the incorporation of glucose modified the surface environment, promoting a redistribution of acidic species and changing their adsorption strength.^42^

A more pronounced effect was observed for HAp.Sac, which exhibited exclusively weak and medium-strength acidic sites, with no detectable contribution from strong acid sites. FTIR analysis did not reveal residual organic species after calcination, indicating that the suppression of strong acid sites cannot be attributed to the presence of carbonaceous residues. Instead, this behavior appears to be associated with structural and textural modifications induced by sucrose during synthesis. BET analysis revealed a substantial increase in specific surface area from 28 m^2^ g^–1^ for HAp.amb to 155 m^2^ g^–1^ for HAp.Sac, along with an increase in pore volume from 0.147 to approximately 0.59 cm^3^ g^–1^. In addition, XRD results showed a decrease in crystallite size from 13.82 to 11.50 nm. These results suggest that sucrose acts as a structure-directing and pore-forming agent, limiting crystal growth and promoting the formation of a more dispersed and porous hydroxyapatite structure. Such structural changes can alter the distribution, accessibility, and strength of surface acid sites, favoring the predominance of medium-strength acidity while suppressing strongly acidic adsorption environments.

The textural properties obtained from BET analysis revealed a significant increase in specific surface area and pore volume for the modified samples. The BET surface area increased from 28m^2^ g^–1^ for HAp.amb to 155 and 125m^2^ g^–1^ for HAp.Sac and HAp.Gli, respectively, while the pore volume increased from 0.147 cm^3^ g^–1^ to approximately 0.59 cm^3^ g^–1^. XRD analysis indicated a reduction in crystallite size to 11.50 nm for HAp.Sac and 12.23 nm for HAp.Gli. These results indicate that sucrose and glucose acted as structure-directing agents during synthesis, restricting crystal growth and promoting the development of a more porous network. Consequently, the enhanced catalytic performance observed for HAp.Sac and HAp.Gli can be attributed to their higher surface areas, larger pore volumes, and smaller crystallite sizes, which increase the accessibility of active sites and facilitate mass transport during the reaction.

Overall, the TPD results indicate that the incorporation of sugars effectively modulates both the strength and distribution of acidic and basic surface sites. While glucose promoted a gradual redistribution of acid–base properties, sucrose induced more significant modifications, resulting in the predominance of medium-strength acid sites and the disappearance of detectable strong acid sites. These changes, combined with the enhanced textural properties of the modified materials, may be advantageous for catalytic and adsorption processes that depend on tailored acid–base characteristics.^45^

The acid–base distributions observed by TPD are therefore consistent with the textural modifications revealed by nitrogen adsorption–desorption analyses. The increase in surface area, pore volume, and dispersion promoted by the organic additives likely enhanced the accessibility of surface functional groups and modified their local coordination environments, leading to the observed redistribution of acidic and basic sites. This synergistic relationship between textural and surface chemical properties may contribute significantly to the improved catalytic performance of the modified hydroxyapatite samples.

### 4.7 Catalytic Activity Results

The catalytic performance of the synthesized hydroxyapatite materials was assessed through product distribution analysis by gas chromatography. Since no calibration factors or internal standards were employed for absolute quantification, the catalytic results are reported as relative product yields, calculated from the normalized chromatographic peak areas according to eq 1.^46,47^

1 $$relative\;product\;yield\left(\%\right)=\frac{A_{i}}{\Sigma \mathrm{Ai}}\times 100\%$$

where *A_i_* corresponds to the chromatographic peak area of the product of interest and ∑*Ai* represents the sum of the peak areas of all detected liquid products. Therefore, the values reported throughout this work represent relative product yields obtained from chromatographic area normalization rather than absolute molar yields.

The relative *n*-butanol yields obtained for the different catalysts are presented in Figure 11. The results indicate that both catalyst composition and reaction time affected product distribution. Among the investigated samples, HAp.Sac exhibited the lowest relative *n*-butanol yield after 3 h of reaction. In contrast, HAp.Gli showed the highest relative *n*-butanol yield, reaching approximately 87% after 2 h. The catalytic conversion of ethanol to *n*-butanol afforded an average yield of 55.10 ± 1.38% (mean ± standard deviation, *n* = 4), with a coefficient of variation of 2.51%.^47,48^

**Figure 11:**
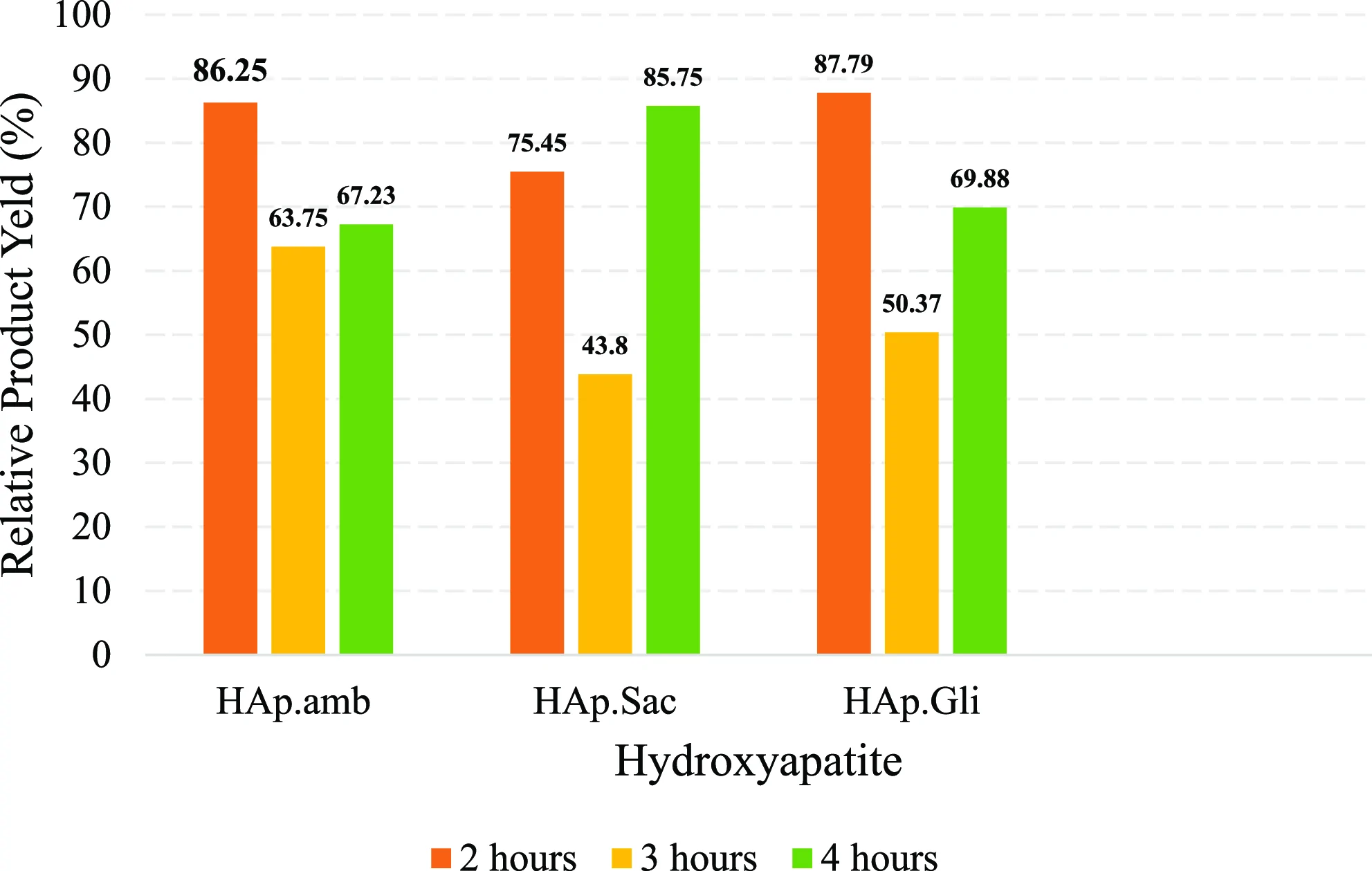
Relative *n*-butanol yield calculated from normalized GC peak areas for pristine HAp, sucrose-modified HAp (HAp.Sac), and glucose-modified HAp (HAp.Gli).

The differences observed among the catalysts can be correlated with the structural and surface characteristics identified by N_2_ adsorption–desorption, FTIR, SEM, and TPD analyses. The HAp.Gli sample exhibited increased mesoporosity together with a more balanced distribution of acidic and basic surface sites. These characteristics may favor ethanol adsorption and the subsequent formation of reaction intermediates, contributing to higher relative *n*-butanol production. Conversely, although HAp.Sac displayed a highly porous structure, its modified acid–base properties may have altered the distribution of active sites required for the reaction, resulting in lower catalytic performance.

The observed catalytic behavior is consistent with the bifunctional nature commonly attributed to hydroxyapatite catalysts. Acidic and basic sites work cooperatively during ethanol upgrading through the Guerbet reaction pathway. Basic sites are generally associated with ethanol activation and dehydrogenation steps, whereas acid–base surface pairs are involved in condensation and C–C bond formation reactions.^49–51^

The superior performance of HAp.Gli is therefore likely related to a favorable combination of textural properties and acid–base functionality. Such characteristics can facilitate the formation and transformation of reaction intermediates involved in the consecutive dehydrogenation, condensation, dehydration, and hydrogenation steps of the Guerbet process.^52–54^ These results suggest that glucose acts as an effective porogenic agent during hydroxyapatite synthesis, promoting physicochemical properties that favor *n*-butanol formation under the reaction conditions evaluated.

The catalytic behavior observed in this study is consistent with the bifunctional character commonly attributed to hydroxyapatite catalysts in the literature. It has been proposed that ethanol upgrading to *n*-butanol occurs through the Guerbet reaction pathway, in which acidic and basic surface sites act cooperatively. In this framework, basic sites are generally believed to promote ethanol activation and dehydrogenation, while acid–base surface pairs may facilitate condensation reactions and subsequent C–C bond formation. Although the present results are consistent with this interpretation, the individual mechanistic steps were not directly investigated in this work.^49–51^ Therefore, the catalytic performance strongly depends on the availability, strength, and distribution of these surface sites.

The enhanced activity observed for HAp.Gli may be attributed to a more favorable balance between textural and acid–base properties, which facilitates the adsorption, diffusion, and transformation of reaction intermediates. Such synergistic effects are particularly important in multistep reactions such as the Guerbet process, where successive dehydrogenation, condensation, dehydration, and hydrogenation reactions occur on the catalyst surface.^52–54^ Consequently, the results indicate that glucose acts as an effective porogenic agent for tailoring hydroxyapatite properties and improving catalytic performance in ethanol upgrading reactions.

## 5 Conclusion

This study demonstrated a clear relationship between synthesis conditions, textural properties, and catalytic performance in hydroxyapatite materials derived from waste eggshells. The incorporation of sucrose and glucose as porogenic agents during the sol–gel synthesis effectively modified the pore structure, resulting in a significant increase in specific surface area, from approximately 28 to 155 m^2^ g^–1^, and the formation of mesoporous architectures with pore diameters ranging from 5 to 35 nm.

Textural characterization revealed that the modified samples developed a more accessible pore network. XRD and FTIR analyses confirmed the preservation of the hydroxyapatite crystalline structure after synthesis and calcination, while TGA, FTIR, and XRD results collectively supported the effective decomposition and removal of the sugar-derived templates during thermal treatment. SEM observations indicated morphological differences among the synthesized materials, whereas the porosity and pore-size distributions were primarily established from BET and BJH analyses.

NH_3_-TPD and CO_2_-TPD analyses showed that the porogenic agents influenced both the density and distribution of acidic and basic surface sites. Among the investigated materials, HAp.Gli exhibited a more balanced acid–base character, with intermediate acidity and the highest density of basic sites. This acid–base profile, combined with enhanced mesoporosity, may contribute to improved reactant accessibility and catalytic performance.

Catalytic evaluation under solvothermal conditions at 180 °C demonstrated that the catalyst composition significantly influenced product distribution. The HAp.Gli catalyst exhibited the highest relative *n*-butanol yield, reaching approximately 87% after 2 h of reaction, based on normalized gas chromatographic peak areas. Although the observed catalytic behavior is consistent with the Guerbet reaction pathway commonly reported for hydroxyapatite-based catalysts, additional mechanistic investigations would be required to confirm the individual reaction steps involved.

Overall, the results indicate that porosity engineering through the use of renewable sugar-based porogenic agents is an effective strategy for tailoring the structural, textural, and surface properties of hydroxyapatite. Furthermore, this work demonstrates the feasibility of producing functional hydroxyapatite catalysts from eggshell waste, providing a sustainable approach for the valorization of an abundant biogenic residue. Describing the synthesis of eggshell-derived hydroxyapatite modified with sugar-based porogenic agents for ethanol upgrading toward *n*-butanol under the conditions investigated.

Although the catalytic results revealed clear differences among the synthesized materials, the experiments were performed as an initial assessment of the catalytic behavior of eggshell-derived hydroxyapatite. Additional replicate catalytic tests, together with quantitative analyses involving calibration factors, internal standards, and carbon balance determination, will be conducted in future studies to evaluate experimental variability and reproducibility. Catalyst recycling and postreaction characterization will also be addressed to assess the stability and reusability of the materials.

These findings provide new insights into the relationship between pore architecture, acid–base surface properties, and catalytic performance in hydroxyapatite materials, demonstrating how simple modifications in the synthesis route can be used to optimize catalyst properties for sustainable catalytic applications.
